# Unraveling the influence of α-mangostin on MDA-MB-231 cell line via WNT/β-catenin signaling pathway: *in silico* and *in vitro* approaches

**DOI:** 10.3389/fphar.2025.1600281

**Published:** 2025-10-01

**Authors:** Riezki Amalia, Citra Dewi, Adryan Fristiohady, Taufik Muhammad Fakih, Muchtaridi Muchtaridi

**Affiliations:** ^1^ Department of Pharmacology and Clinical Pharmacy, Faculty of Pharmacy, Universitas Padjadjaran, Bandung, Indonesia; ^2^ Laboratory of Translational Pharmaceutical Research, Faculty of Pharmacy, Universitas Padjadjaran, Bandung, Indonesia; ^3^ Department of Pharmaceutical Analysis and Medical Chemistry, Faculty of Pharmacy, Universitas Padjadjaran, Bandung, Indonesia; ^4^ Pharmacy Study Program, Faculty of Science and Technology, Universitas Mandala Waluya, Kendari, Indonesia; ^5^ Pharmacy Study Program, Faculty of Pharmacy, Universitas Halu Oleo, Kendari, Indonesia; ^6^ Department of Pharmacy, Faculty of Mathematics and Natural Sciences, Universitas Islam Bandung, Bandung, Indonesia; ^7^ Research Collaboration Centre for Theranostic Radio Pharmaceuticals, National Research and Innovation Agency (BRIN), Bandung, Indonesia

**Keywords:** triple negative breast cancer, α-mangostin, Wnt/β-catenin, GSK-3β, *CCND1*, *MYC*

## Abstract

The Wnt/β-catenin signaling pathway is critically involved in breast cancer progression, particularly in the triple-negative subtype (TNBC). Aberrant activation of this pathway promotes tumor proliferation, with β-catenin functioning as a central effector regulated by GSK-3β-mediated phosphorylation and degradation. Despite its therapeutic significance, no selective Wnt/β-catenin inhibitors have been clinically approved, underscoring the need for alternative strategies. Natural compounds such as α-mangostin have emerged as potential modulators of this pathway. This study investigates the potential of α-mangostin, a natural xanthone compound, to suppress Wnt/β-catenin signaling through complementary *in silico* approaches examining its interaction with proteins related to the Wnt signaling pathway, followed by *in vitro* validation using the MDA-MB-231 triple-negative breast cancer cell line (ER-/PR-/HER2-). In parallel, MCF-7 cells (ER+/PR+/HER2-) were used as a comparator to evaluate the differential inhibitory effects on breast cancer cells with distinct hormonal profiles. Molecular docking demonstrated favorable binding of α-mangostin to β-catenin and LRP6, with higher affinity toward LRP6. Molecular dynamics simulations confirmed the stability of these complexes, particularly the α-mangostin-LRP6 complex, which exhibited minimal RMSD and SASA fluctuations. Consistently, MM/PBSA calculations revealed the most favorable binding free energy for α-mangostin with LRP6 (−96.659 kJ/mol). *In vitro* WST-8 assays revealed that α-mangostin reduced cell viability in both cell lines, with a greater suppressive effect observed in combination with LiCl. Treatment with 10 µM α-mangostin, alone or with LiCl, significantly downregulated the Wnt transcriptional targets *CCND1* (5.2-fold) and *MYC* (3.3-fold) in MDA-MB-231 cells, as determined by RT-qPCR, thereby indicating a potent suppressive effect on the Wnt pathway. Collectively, these findings indicate that α-mangostin exerts anticancer effects by targeting multiple components of the Wnt/β-catenin pathway, with LRP6 emerging as its primary target. Further investigations are warranted to elucidate its impact on β-catenin phosphorylation and to validate its efficacy *in vivo*.

## 1 Introduction

According to data from the Global Burden of Cancer Study (GLOBOCAN) by the World Health Organization (WHO) in 2020, breast cancer exhibits a high incidence rate in Asia, accounting for 45.5% of cases and contributing to 50.5% of cancer-related deaths (Sung et al., 2021). In Indonesia specifically, breast cancer ranks highest among new cases, with 65,858 instances, representing 16.6% of the total 396,914 reported cancer cases (Andinata et al., 2023). Among the breast cancer subtypes, triple-negative breast cancer (TNBC) is known for its poor prognosis. TNBC has limited treatment options compared to other breast cancer subtypes (Chaudhuri et al., 2022; Lee, 2023). The aggressive nature of TNBC underscores the urgency for targeted therapies that can effectively manage its progression and improve patient outcomes.

TNBC is characterized by the absence of estrogen receptor, progesterone receptor, and Human Epidermal Receptor-2 (HER-2) expression (Treeck et al., 2020). This type of cancer is more prevalent in individuals with *BRCA1*/*BRCA2* gene mutations, which predispose them to cancer development (Mehrgou and Akouchekian, 2016). Alterations influence cancer progression in multiple signaling pathways, including the Wnt/β-catenin pathway. Various target genes of the Wnt signaling pathway have been identified and are implicated in regulating processes such as cell proliferation, invasion, metastasis, apoptosis, and resistance to chemotherapy (Zhang and Wang, 2020). The Wnt signaling pathway encompasses two main branches: canonical and non-canonical pathways, each playing distinct roles in cellular function and cancer progression (Liu et al., 2022). Understanding the intricate mechanisms of Wnt signaling in TNBC could lead to novel therapeutic strategies targeting this aggressive subtype of breast cancer.

The canonical Wnt pathway initiates with the binding of the Wnt ligand to the receptor complex composed of frizzled (FZD) and low-density lipoprotein receptor-related protein 5/6 (LRP5/6) (Jeong and Jho, 2021). This binding event prevents GSK-3β from phosphorylating β-catenin, thereby inhibiting its ubiquitination and degradation (Lin et al., 2020). Consequently, β-catenin accumulates in the cytoplasm and translocates into the nucleus, where it binds to TCF/LEF transcription factors and stimulates the transcription of target genes such as *CCND1* (*Cyclin D1*), *MYC (c-Myc)*, *AXIN2*, and *BIRC5* (*Survivin*) (Liu et al., 2022; Pai et al., 2017). Additionally, the Wnt/β-catenin signaling pathway plays a crucial role in TNBC, with β-catenin, Axin, and APC being key players in this pathway. Aberrant activation of this pathway, often observed in TNBC, contributes to cell proliferation, tumor development, progression, and metastasis, making it a potential therapeutic target for this aggressive form of breast cancer. Part of the destruction complex that regulates β-catenin levels. In the absence of Wnt signaling, AXIN, along with APC and GSK-3β, promotes the phosphorylation and degradation of β-catenin. However, when Wnt signaling is activated, AXIN’s function is inhibited, leading to β-catenin stabilization and nuclear translocation. Another critical component of the destruction complex. APC binds to β-catenin and promotes its degradation. Mutations in APC can lead to the stabilization of β-catenin and aberrant Wnt signaling (Jeong and Jho, 2021; Wu et al., 2025). Elucidating these molecular mechanisms is crucial not only for understanding cancer pathogenesis but also for developing targeted therapies that can potentially inhibit Wnt signaling in cancer.

Several synthetic Wnt inhibitors have been developed, each targeting different components of the pathway. For instance, LGK974 blocks the secretion of porcupine, a Wnt-acyltransferase (Liu et al., 2013), while ICG-001 and its derivative PRI-724 disrupt the β-catenin/CBP interaction, thereby inhibiting downstream transcriptional activity in head-and-neck squamous carcinoma and pancreatic cancer cells. These inhibitors are currently undergoing preclinical and clinical evaluation across various cancer types (Kimura et al., 2022). In addition to synthetic compounds, numerous natural products have demonstrated potential in modulating Wnt signaling. For example, curcumin, resveratrol, and epigallocatechin gallate (EGCG) have been reported to inhibit Wnt/β-catenin signaling through mechanisms such as downregulation of β-catenin expression or interference with its nuclear translocation (Ashrafizadeh et al., 2020; Vallée et al., 2019; Yang et al., 2016). Owing to their multitarget effects and relatively low toxicity, these natural agents represent promising adjunctive strategies for Wnt-targeted cancer therapy.

A natural compound recognized for its anti-breast cancer properties is α-mangostin (Muchtaridi et al., 2019; Nalla and Ganta, 2023; Nurhidayah et al., 2023; Sarmoko et al., 2023). Research has shown that α-mangostin effectively inhibits proliferation and induces apoptosis in SKBR3, MCF-7, and MDA-MB-231 cells, with IC_50_ values of 9.69 μM, 11.37 μM, and 7.46 μM, respectively (Zhu et al., 2021). The Wnt-mediated modulation of α-mangostin’s anticancer activity has been reported in both osteosarcoma and colon cancer cells. A recent study by Yang S. et al. (2021) demonstrated that α-mangostin induces endoplasmic reticulum (ER) stress due to excessive reactive oxygen species (ROS) generation, leading to the inactivation of the Wnt signaling pathway and subsequently triggering apoptosis through caspase cleavage (Yang S. et al., 2021). In addition, an earlier investigation by Yoo et al. (2011) in colon cancer cells found that α-mangostin inhibits TCF/β-catenin transcriptional activity and downregulates β-catenin protein levels. However, the stability of β-catenin remains unaffected (Yoo et al., 2011). Despite these findings, no study to date has evaluated the effect of α-mangostin on the expression of Wnt/β-catenin target genes in TNBC. Therefore, the present study aims to investigate the therapeutic potential of α-mangostin in breast cancer, focusing specifically on its effects on the Wnt signaling pathway through both *in vitro* and *in silico* approaches. *In vitro* experiments investigated the inhibitory effects of α-mangostin on MDA-MB-231 and MCF-7 cells under conditions of Wnt/β-catenin pathway activation. To evaluate the inhibition of breast cancer cell proliferation, MCF-7 cells (ER+/PR+/HER2−, luminal subtype) were used as a comparator against MDA-MB-231 cells (triple-negative, ER−/PR−/HER2−). Gene expression levels of *CCND1* and *MYC* were quantified using RT-qPCR to assess the downstream impact of α-mangostin on Wnt pathway target genes involved in cell proliferation and survival in MDA-MB-231 cells. Concurrently, *in silico* methods will be employed to elucidate the interaction between α-mangostin and key components of the Wnt/β-catenin signaling pathway, including LRP6 and β-catenin. This integrated approach aims to provide comprehensive insights into the therapeutic potential of α-mangostin in targeting the Wnt/β-catenin pathway and its implications for breast cancer treatment, notably in TNBC.

## 2 Materials and methods

### 2.1 *In silico* approaches

#### 2.1.1 Molecular docking simulation

The initial structures for molecular modeling were obtained from the crystal structures of β-catenin (PDB ID: 2GL7, resolution 2.60 Å) and LRP6 (PDB ID: 3S2K, resolution 2.80 Å) (Ahn et al., 2011; Sampietro et al., 2006) (Table 1). Missing loop regions were reconstructed using the *auto model* and *loop model* functions of MODELER in Discovery Studio (DS) 2019 Client (Kemmish et al., 2017). Prior to molecular docking simulations, water molecules and ligands were removed, and hydrogen atoms were added to the protein structures. Further preparation of β-catenin and LRP6 was carried out using UCSF Chimera and AutoDockTools 4.2.6, with AD4 atom types and Gasteiger charges assigned to optimize docking accuracy (Forli et al., 2016; Pettersen et al., 2004).

**TABLE 1:** Docking parameters for β-Catenin and LRP6 binding sites.

Protein binding site	Grid center (x, y, z)	Grid box size (Å)	Spacing (Å)	Search exhaustiveness
β-catenin US	11.527 × 22.308 × 62.347	64 × 60 × 60	0.375	100
β-catenin AS	2.805 × 14.864 × 79.543	64 × 60 × 60	0.375	100
LRP6 E3	26.038 × 5.167 × −15.27	64 × 60 × 60	0.375	100

β-Catenin possesses two primary binding sites: the canonical β-catenin union site (β-catenin US), which includes Asp16 from TCF4, Lys435, and His470, with the NZ atom of Lys435 defined as the grid center; and an allosteric site (β-catenin AS), characterized by Pro521, Arg528, and Asp583, with the ND1 atom of His524 designated as the center. For LRP6, the docking grid was centered at Gly227 from DKK1, consistent with previously reported binding interactions. Initial docking poses for subsequent molecular dynamics (MD) simulations were generated using AutoDockTools 4.2.6, with docking parameters optimized according to a previous study (Vélez-Vargas et al., 2023). The best binding poses were selected based on binding affinity and further validated by visual inspection using Discovery Studio (DS) 2019 Client, PLIP, and UCSF Chimera. For comparative analysis, hit derivatives were also evaluated using Schrödinger’s Glide software (version 2019–1, Schrödinger, LLC, New York, NY, United States, 2017) (Durrant and McCammon, 2011). These docking results provided the foundation for further MD simulations, enabling a detailed These docking results served as the basis for MD simulations, allowing detailed investigation of the stability and interactions of α-mangostin and its derivatives with β-catenin and LRP6.

#### 2.1.2 Molecular dynamics (MD) simulation

All-atom molecular dynamics (MD) simulations of the top-ranked docking poses were performed using GROMACS 2016.3 with the PLUMED 2.4 plugin (Abraham et al., 2015). Protein and ligand parameters were generated using the CHARMM36 force field and CGenFF (Wurl and Ferreira, 2023), with systems solvated in a TIP3P water box (10 Å buffer) and neutralized with 0.15 M NaCl. Energy minimization was performed using the steepest descent method until a tolerance of 1000 kJ/mol was achieved, followed by NVT equilibration (25 ps, 303.15 K). NPT production runs for 500 ns at 303.15 K and 1 bar, controlled by the Nosé–Hoover thermostat and Parrinello–Rahman barostat (Shiga et al., 2023). Bond constraints involving hydrogen atoms were applied using the LINCS algorithm (Hess et al., 1997). The production runs employed a 2 fs timestep with trajectories saved every 1 ps; electrostatic interactions were treated with PME, and van der Waals interactions were truncated at 12 Å. An upper-wall restraint (200 kJ/mol·nm^-2^) was applied when the ligand center of mass exceeded 12 Å from the binding site. Protein–ligand interactions were analyzed over the final 200 ns using GROMACS 2016.3, Discovery Studio 2019 (Kemmish et al., 2017), and VMD 1.9.4 (Humphrey et al., 1996).

### 2.2 *In vitro* evaluation

#### 2.2.1 Cell culture

MDA-MB-231 and MCF-7 cell lines were obtained from the European Collection of Authenticated Cell Cultures (ECACC; MDA-MB-231, catalog no. 92020424; MCF-7, catalog no. 86012803) and maintained at the Translational Pharmaceutical Research Laboratory, Faculty of Pharmacy, Universitas Padjadjaran, Sumedang, Indonesia.

MDA-MB-231 cells were maintained at 37 °C in Dulbecco’s Modified Eagle’s Medium (DMEM) supplemented with 15% fetal bovine serum (FBS), 2 mM L-glutamine, 100 IU/mL penicillin, and 10 μg/mL streptomycin in a humidified atmosphere containing 5% CO_2_. The cells reached optimal growth at a density of 1–3 × 10^4 cells/cm^2^. Once confluent, cultures were harvested by adding 1–2 mL of 0.25% trypsin. The detached cells were transferred to a conical tube, neutralized with fresh DMEM containing FBS to a final volume of 10 mL, and centrifuged at 2000 rpm for 5 min. The supernatant was discarded, and the resulting pellet was resuspended in 1 mL of medium. Viable cells were then counted using a hemocytometer. MCF-7 cells were cultured following the same procedure, except that Eagle’s Minimum Essential Medium (EMEM, EBSS) supplemented with 2 mM L-glutamine, 1% non-essential amino acids (NEAA), and 10% FBS was used.

#### 2.2.2 Cell proliferation inhibitor assay

Cell proliferation was assessed using the WST-8 assay. MDA-MB-231 and MCF-7 cells were seeded into 96-well plates and treated for 24 h with α-mangostin (60, 120, 240, and 480 µM), LiCl (2.5, 5, and 10 mM) or their combinations. After treatment, cells were washed with PBS and incubated with 100 µL of 0.5 mg/mL WST-8 solution at 37 °C for 2–4 h. Absorbance was measured at 450 nm using a Tecan Infinite spectrophotometer, and cell viability was calculated relative to untreated controls.

#### 2.2.3 Expression quantification of *CCND1* and *MYC*

The MDA-MB-231 cell line was seeded into 6-well plates and incubated for 24 h. Cells were then treated with α-mangostin at concentrations of 5 μM and 10 µM or with cisplatin at 1.25 µM and 2.5 µM, either alone or in combination with LiCl (5 mM). RNA was isolated using the GeneZol™ Kit according to the manufacturer’s instructions, and the resulting RNA was resuspended in nuclease-free water (NFW). RT-qPCR was performed with the SensiFAST™ SYBR^®^ No-ROX One-Step Kit in a total reaction volume of 20 μL. The amplification program consisted of an initial denaturation at 95°C for 5 s, followed by 40 cycles of 95 °C for 30 s, 55 °C for 60 s, and 72 °C for 60 s. Primers specific to the target genes are listed in Table 2, with *GAPDH* used as the housekeeping control gene for normalization (Sun et al., 2015; Xiang et al., 2021). Relative gene expression was calculated using the 2^−ΔΔCT^ method, and all experiments were conducted in biological triplicates (n = 3).

**TABLE 2:** Primer sequences of target genes and the *GAPDH* reference gene for RT-qPCR analysis.

Gene	Primer	Annealing temperature
CCND1	F:5′-TAGATGCACAGCTTCTCGGC-3′ R:5′-CTGCGAAGTGGAAACCATCC-3′	60 °C
MYC	F:5′-CCTCGGATTCTCTGCTCTCC-3′ R:5′-TTTCTTCCTCATCTTCTTGTTCCTC-3′	58 °C
*GAPDH*	F:5′-CACCCACTCCTCCACCTTTG-3′ R:5′-CCACCACCCTGTTGCTGTAG-3′	60 °C

#### 2.2.4 Statistical analyses

Statistical analyses were performed using Microsoft Excel and GraphPad Prism 10.0.0 (GraphPad Software). All data are expressed as mean ± SD. Differences between groups were analyzed using one-way ANOVA followed by Tukey’s *post hoc* test, with significant p-values indicated in the figures. Significance levels are indicated as follows: P < 0.05; *P < 0.01; and **P < 0.001.

## 3 Results

### 3.1 *In silico* approach

#### 3.1.1 Dynamic molecular behavior and system stability

**FIGURE 1:**
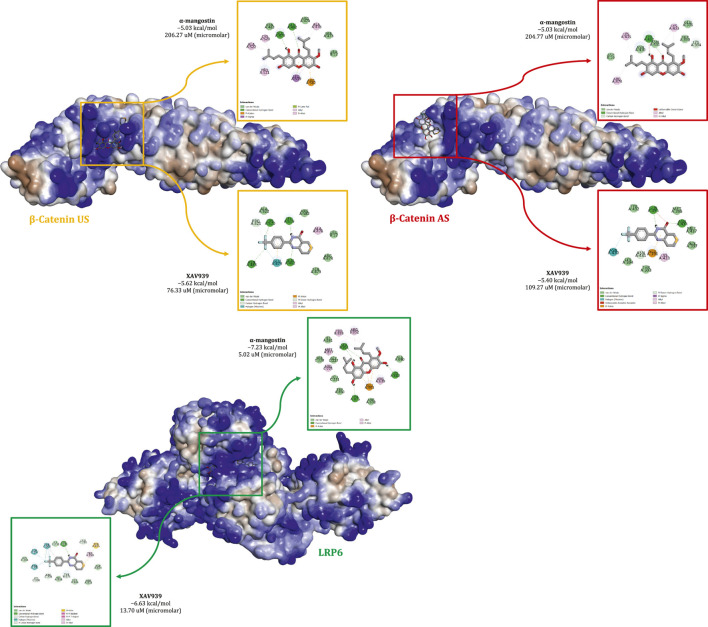
Comparative binding interactions of α-mangostin and XAV939 with β-catenin (union site, US; allosteric site, AS) and LRP6, showing docking affinities, key molecular interactions, and electrostatic surface representations.

Previous findings suggest that α-mangostin exerts antiproliferative effects in MDA-MB-231 cells by modulating the transcription of key Wnt-regulated genes involved in proliferation. To further elucidate the molecular basis of this effect, *in silico* analyses were performed to investigate the interaction of α-mangostin with β-catenin and LRP6. Figure 1 shows the binding profiles of α-mangostin and XAV939 within the β-catenin union site (US), the allosteric site (AS), and the LRP6 receptor. The surface representations highlight the spatial orientation and electrostatic characteristics of the binding pockets, providing context for the stability of interactions. At the β-catenin US, α-mangostin exhibited a docking score of −5.03 kcal/mol with a relatively weak binding affinity (Kd = 206.27 μM). By comparison, XAV939 demonstrated slightly stronger binding at −5.62 kcal/mol with a Kd of 76.33 μM, suggesting better accommodation within the same pocket. The β-catenin AS displayed similar energetics, with α-mangostin docking at −5.03 kcal/mol (Kd = 204.77 μM) and XAV939 at −5.40 kcal/mol (Kd = 109.27 μM), consistent with the more solvent-exposed and structurally relaxed nature of this site. In contrast, α-mangostin exhibited substantially stronger binding to LRP6, with a docking score of −7.23 kcal/mol and a low dissociation constant of 5.02 μM, indicating high affinity. XAV939 also bound LRP6 with favorable energetics (ΔG = −6.63 kcal/mol, Kd = 13.70 μM), but with lower affinity compared to α-mangostin. The binding orientation of α-mangostin in LRP6 was more deeply encapsulated, suggesting enhanced shape complementarity. Electrostatic surface mapping further revealed that LRP6 provides superior charge compatibility, facilitating stronger non-covalent interactions, including hydrogen bonding and π-interactions. Taken together, these results indicate that LRP6 serves as a more selective and energetically favorable receptor for α-mangostin. The improved structural anchoring in LRP6 supports its potential for greater biological efficacy and retention, which may be critical for downstream pharmacological activity (Medina-Barandica et al., 2023).

**FIGURE 2:**
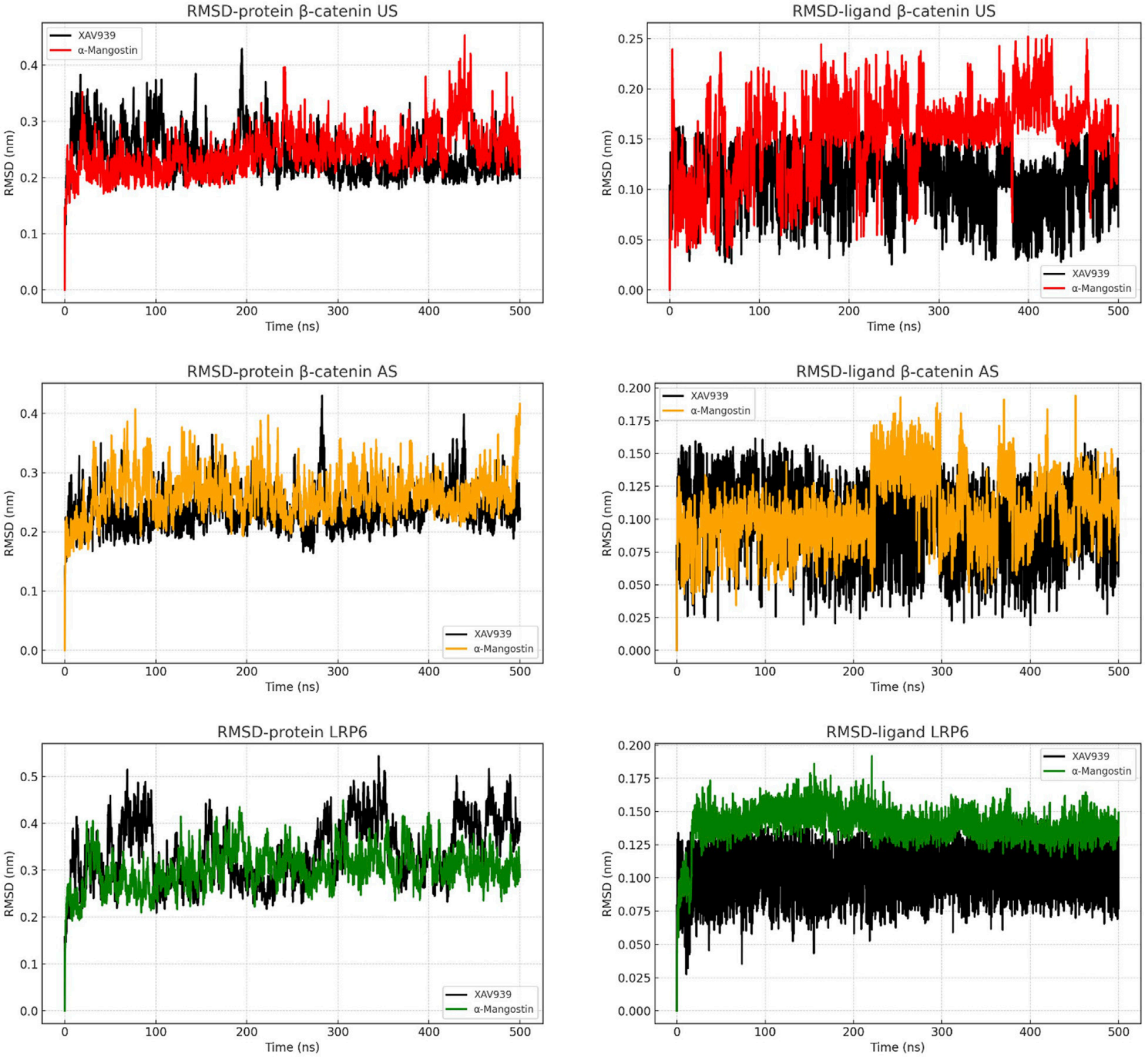
RMSD analysis of backbone atoms and ligand dynamics in β-catenin (union site, US; allosteric site, AS) and LRP6 throughout the simulation time.

Structural stability of the protein–ligand complexes was assessed by root mean square deviation (RMSD) analysis (Figure 2). In the β-catenin US complex, α-mangostin induced a gradual increase in protein RMSD, exceeding 0.35 nm toward the end of the simulation, whereas XAV939 maintained greater stability with values below 0.30 nm. Ligand RMSD plots showed that α-mangostin fluctuated around 0.20–0.22 nm, while XAV939 remained slightly lower at 0.15–0.20 nm. The β-catenin AS complex demonstrated improved stability, with both ligands maintaining consistent protein RMSD values between 0.25 and 0.30 nm. Ligand fluctuations in AS were also reduced compared to US, although α-mangostin still exhibited slightly higher deviation than the reference ligand. By contrast, LRP6 complexes exhibited the highest stability, with protein RMSD values consistently within the range of 0.20–0.30 nm throughout the simulation. Ligand RMSDs in LRP6 remained particularly low, with α-mangostin deviating by less than 0.15 nm. These findings indicate that LRP6 provides a more rigid and stable binding environment compared to β-catenin. The stable RMSD patterns observed in LRP6 suggest minimal structural rearrangements during ligand binding. Overall, these results highlight LRP6 as a conformationally stable and favorable target, supporting its potential for further drug development under physiological conditions (Aulifa et al., 2024).

**FIGURE 3:**
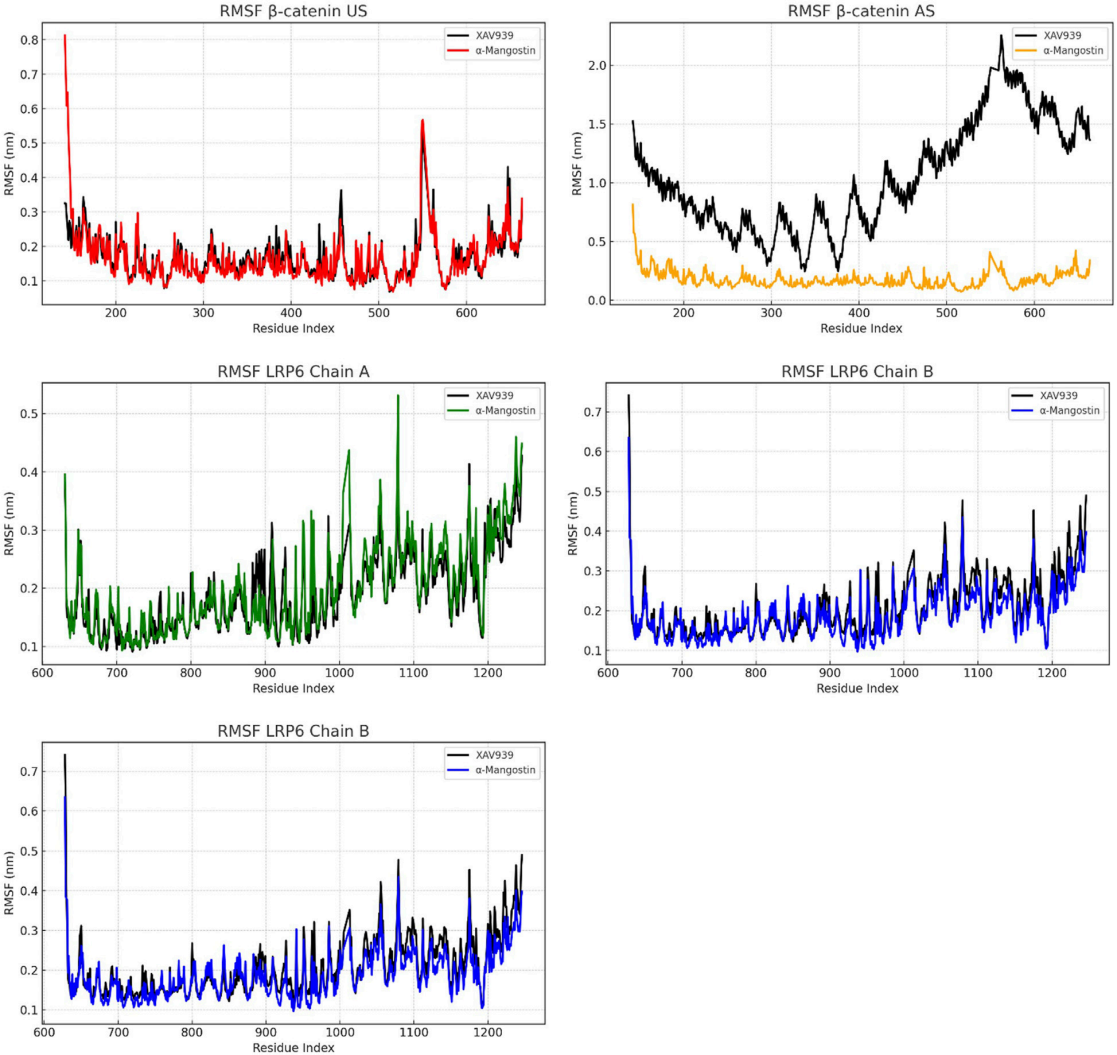
RMSF analysis of β-catenin (union site, US; allosteric site, AS) and LRP6, showing residue-level flexibility and solvent-exposed interactions.

To examine residue-level dynamics, root mean square fluctuation (RMSF) analyses were performed, as shown in Figure 3. In the β-catenin US complex, RMSF values for both ligands remained below 0.5 nm, with α-mangostin producing lower fluctuations than XAV939 across several regions. A minor peak was observed near residue index 600 for both ligands, indicating localized flexibility. In the AS configuration, the pattern changed markedly, as XAV939 displayed prominent peaks exceeding 2.0 nm, while α-mangostin exhibited restrained movements, with most residues fluctuating by less than 0.5 nm. These results suggest that α-mangostin stabilizes local dynamics more effectively in the AS. For LRP6, residue fluctuations were even more controlled, particularly in chain A, where RMSF values ranged between 0.2 and 0.4 nm. Chains B and C demonstrated similarly low fluctuations, with α-mangostin yielding slightly smoother profiles and values consistently below 0.3 nm. Across all chains, α-mangostin reduced residue mobility more effectively than the reference ligand. Reduced flexibility at the binding site contributes to enhanced ligand residency and complex stability. Overall, these RMSF trends support the conclusion that LRP6 provides a more rigid and stable interaction interface compared to β-catenin, a property advantageous for the design of ligands targeting dynamic protein systems.

**FIGURE 4:**
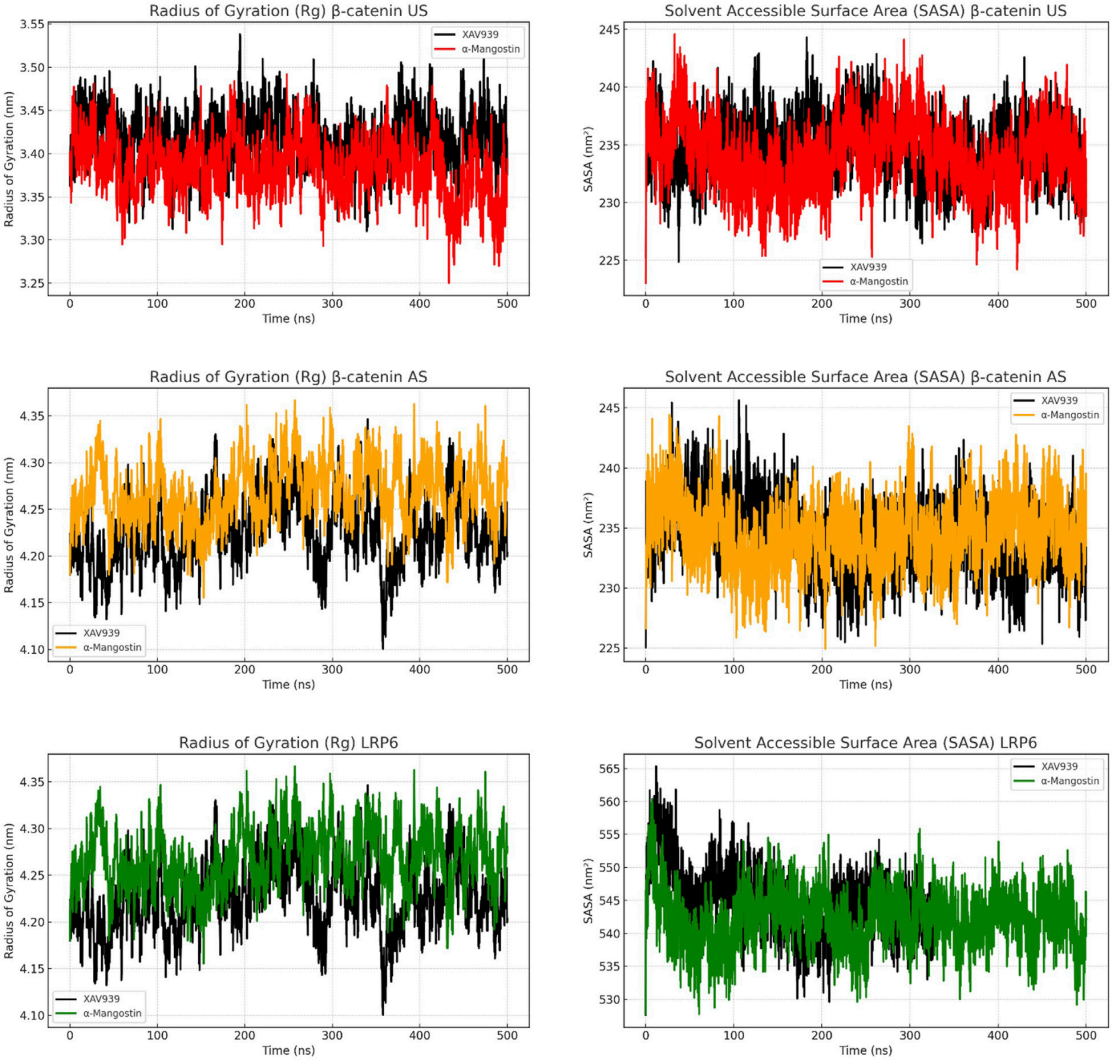
Figure 6. Radius of gyration (Rg) and solvent-accessible surface area (SASA) analyses of β-catenin (union site, US; allosteric site, AS) and LRP6 following molecular dynamics simulations, evaluating structural compactness and solvent exposure over time.

Figure 4 presents the radius of gyration (Rg) and solvent-accessible surface area (SASA) analyses to evaluate protein compactness and surface exposure. In the β-catenin US complex, Rg values for α-mangostin fluctuated between 3.30 and 3.50 nm, slightly higher than those observed with XAV939. SASA plots indicated that α-mangostin increased solvent exposure, reaching approximately 240 nm^2^. In the AS complex, both ligands exhibited higher Rg values, up to 4.35 nm, while SASA values remained comparable, suggesting moderate surface breathing and reduced folding compactness. By contrast, LRP6 demonstrated tighter Rg distributions (4.10–4.30 nm) and consistently lower SASA values compared with the β-catenin systems. α-Mangostin further reduced SASA in LRP6 relative to XAV939, reflecting deeper ligand embedding within the protein pocket. Compact protein structures are generally associated with enhanced energetic stability and resistance to denaturation or degradation (Buchberger et al., 2010). while reduced solvent exposure favors stronger hydrophobic interactions and improved binding efficiency. Collectively, the Rg and SASA results confirm that LRP6 maintains a more compact and tightly folded conformation during simulation, consistent with earlier findings on backbone and residue-level dynamics. Moreover, α-mangostin contributes to sustaining protein compactness in LRP6, reinforcing its potential as a stable binder.

**FIGURE 5:**
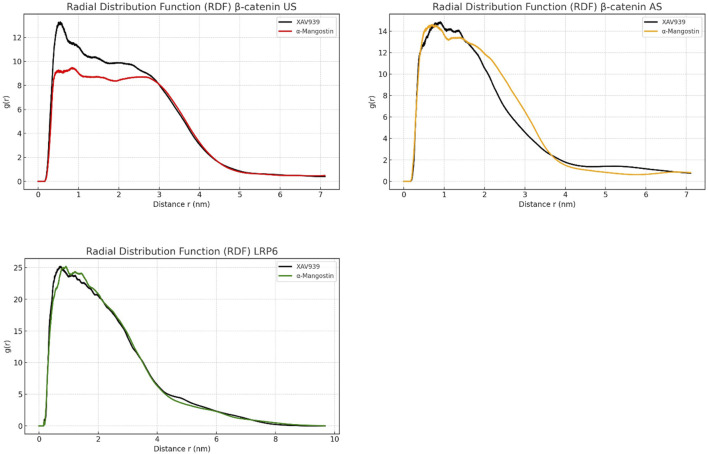
Radial distribution function (RDF) analysis of β-catenin (union site, US; allosteric site, AS) and LRP6 following molecular dynamics simulations, illustrating atomic-level spatial distribution around the ligand-binding regions.

Hydration behavior around the ligand-binding regions was evaluated using radial distribution function (RDF) analysis, as shown in Figure 5. RDF describes the spatial arrangement of solvent molecules in the vicinity of ligands. In the β-catenin US complex, both ligands exhibited broad and less intense RDF peaks, suggesting a disordered solvation shell. XAV939 reached a maximum g(r) value of approximately 13, whereas α-mangostin was slightly lower at around 12. The β-catenin AS complex displayed sharper peaks, although still less structured compared with LRP6. In this system, XAV939 peaked at g(r) = 14, while α-mangostin remained lower, indicating that α-mangostin generates a less disrupted hydration environment. By contrast, LRP6 showed the most structured hydration profile, with sharp peaks centered at 0.5–0.6 nm. Notably, α-mangostin reached a g(r) value close to 25, significantly higher than XAV939. These results suggest that LRP6 forms a compact and stable hydration shell around the ligand. A well-ordered water layer enhances molecular rigidity and contributes favorable enthalpic interactions, thereby influencing ligand stability and retention within the binding pocket. Collectively, the RDF patterns support the conclusion that LRP6 provides a more dynamically favorable environment, consistent with the RMSD and RMSF analyses, which demonstrate the stability of LRP6–ligand complexes.

Altogether, the simulation results provide a cohesive view of the structural behavior of β-catenin and LRP6 when bound to XAV939 and α-mangostin. β-Catenin, particularly in its AS binding configuration, exhibited greater flexibility and higher solvent exposure, which may limit its suitability as a stable target for inhibitors (Hankey et al., 2018). Although the US configuration showed somewhat improved stability, it still demonstrated more fluctuations compared with LRP6. Across all structural metrics evaluated (RMSD, RMSF, RDF, Rg, and SASA), LRP6 consistently outperformed β-catenin, indicating that LRP6 is a structurally rigid and solvent-protected receptor well suited for ligand interactions (Raisch et al., 2019). α-Mangostin performed favorably by stabilizing both protein and solvent dynamics, particularly within the LRP6 binding pocket, suggesting its potential role as a multi-site inhibitor of Wnt signaling. Given that β-catenin regulates downstream oncogenic pathways, especially in colorectal and breast cancers. At the same time, LRP6 functions as a co-receptor in the upstream Wnt complex; targeting both proteins could provide synergistic suppression of Wnt signaling. Previous studies have also shown that LRP6-targeted agents reduce cancer proliferation with fewer off-target effects. The consistent molecular stability observed in this study further supports α-mangostin’s candidacy for development as a Wnt pathway inhibitor (Ariyanto et al., 2023). Nevertheless, additional *in vitro* and *in vivo* studies are warranted to validate these simulation-based insights.

#### 3.1.2 Mechanism of binding interaction as deduced from calculations of binding free energy

The MM/PBSA method was applied to estimate the binding free energies of α-mangostin and XAV939 with β-catenin (US and AS) and LRP6, providing insights into the stability of the complexes. As shown in Table 3, the calculated ΔG_bind values for α-mangostin were −76.437 kJ/mol at the β-catenin US site, −76.167 kJ/mol at the AS site, and −96.659 kJ/mol with LRP6. These results indicate that LRP6 forms the most energetically favorable complex with α-mangostin. The electrostatic contribution (ΔE_elec) was particularly significant in the LRP6–α-mangostin complex (−53.275 kJ/mol), whereas the AS site showed negligible electrostatics (0.042 kJ/mol). Van der Waals interactions (ΔE_vdW) followed a similar pattern, being strongest in the LRP6 complex (−187.046 kJ/mol). These findings suggest that LRP6 provides a deeply hydrophobic and structurally complementary binding pocket for α-mangostin. The gas-phase interaction energy (ΔG_gas), which combines ΔE_vdW and ΔE_elec, was lowest for the LRP6 complex (−19.664 kJ/mol), further supporting its thermodynamic advantage. Although LRP6 showed the highest desolvation penalty (ΔG_solv = 163.326 kJ/mol), this was offset by its favorable gas-phase and non-covalent interactions. In comparison, the β-catenin US and AS sites demonstrated similar ΔG_bind values, with weaker electrostatic contributions in the AS site balanced by stronger hydrophobic interactions. Collectively, the MM/PBSA results confirm that α-mangostin exhibits a stronger binding affinity for LRP6 than for β-catenin.

**TABLE 3:** Binding energy profiles of α-mangostin and native ligand at target binding domains.

Energy components					
Complex	ΔE_vdW	ΔE_elec	ΔG_solv	ΔG_gas	ΔG_bind
β-catenin US + α-mangostin	−95.107 ± 16.696 kJ/mol	−12.097 ± 15.115 kJ/mol	41.198 ± 70.879 kJ/mol	−10.431 ± 2.039 kJ/mol	−76.437 ± 71.461 kJ/mol
β-catenin AS + α-mangostin	−136.361 ± 15.153 kJ/mol	0.042 ± 10.173 kJ/mol	74.023 ± 15.896 kJ/mol	−13.871 ± 1.468 kJ/mol	−76.167 ± 14.679 kJ/mol
LRP6 α-mangostin	−187.046 ± 10.710 kJ/mol	−53.275 ± 10.022 kJ/mol	163.326 ± 16.587 kJ/mol	−19.664 ± 0.984 kJ/mol	−96.659 ± 12.200 kJ/mol
β-catenin US + XAV939	−70.669 ± 27.911 kJ/mol	−9.210 ± 10.714 kJ/mol	49.112 ± 34.260 kJ/mol	−8.162 ± 3.023 kJ/mol	−38.929 ± 19.234 kJ/mol
β-catenin AS + XAV939	−48.478 ± 41.830 kJ/mol	−4.988 ± 8.937 kJ/mol	33.111 ± 33.121 kJ/mol	−5.718 ± 4.975 kJ/mol	−26.072 ± 29.580 kJ/mol
LRP6 XAV939	−145.999 ± 13.009 kJ/mol	−28.658 ± 7.581 kJ/mol	128.930 ± 11.327 kJ/mol	−15.197 ± 0.684 kJ/mol	−60.925 ± 15.305 kJ/mol

A similar trend was observed with the reference ligand XAV939, although its ΔG_bind values were consistently less negative than those of α-mangostin. XAV939 bound to the β-catenin US with a ΔG_bind of −38.929 kJ/mol and to the AS with −26.072 kJ/mol, indicating relatively weak complex formation. Its interaction with LRP6 was more favorable (ΔG_bind = −60.925 kJ/mol) but remained notably less negative than that of the α-mangostin–LRP6 complex. In all comparisons, α-mangostin demonstrated stronger binding, particularly with LRP6, thereby reinforcing its potential as a superior inhibitor scaffold for modulating the Wnt/β-catenin pathway. The strong electrostatic and hydrophobic contributions observed in the α-mangostin–LRP6 complex suggest robust anchoring and reduced dissociation, which may support prolonged biological activity. These findings are consistent with structural dynamics results, including the low RMSF and stable RDF profiles observed in LRP6 simulations. The pronounced ΔG_solv further underscores the role of hydrophobic stabilization within the binding pocket. The comparable ΔG_bind values at the β-catenin US and AS imply that both regions remain accessible targets, albeit thermodynamically less favorable than those at LRP6. Collectively, LRP6’s superior energetic profile highlights it as a more attractive therapeutic target. These binding energy trends provide a rationale for ligand optimization strategies aimed at strengthening van der Waals and electrostatic interactions. Experimental validation using biophysical approaches such as isothermal titration calorimetry (ITC) or surface plasmon resonance (SPR) will be essential to confirm these computational predictions.

### 3.2 Inhibitory effect of α-mangostin on the proliferation of MDA-MB-231 and MCF-7 breast cancer cells

We evaluated the antiproliferative effects of α-mangostin on two distinct subtypes of breast cancer: triple-negative (MDA-MB-231, ER−/PR−/HER2−) and luminal (MCF-7, ER+/PR+/HER2−). During the experiment, we co-treated the cells with lithium chloride (LiCl), an agonist of the canonical Wnt signaling pathway, which inhibits GSK-3β activity and effectively stabilizes free cytosolic β-catenin in cancer cells (Sun and Liu, 2017). In this study, LiCl was applied at concentrations of 2.5, 5, and 10 mM as a control. Previous studies have shown that low concentrations of LiCl (around 4 mM) can promote mesenchymal stem cell proliferation, whereas higher concentrations (20–40 mM) exert inhibitory effects on cell proliferation (De Boer et al., 2004).

**FIGURE 6:**
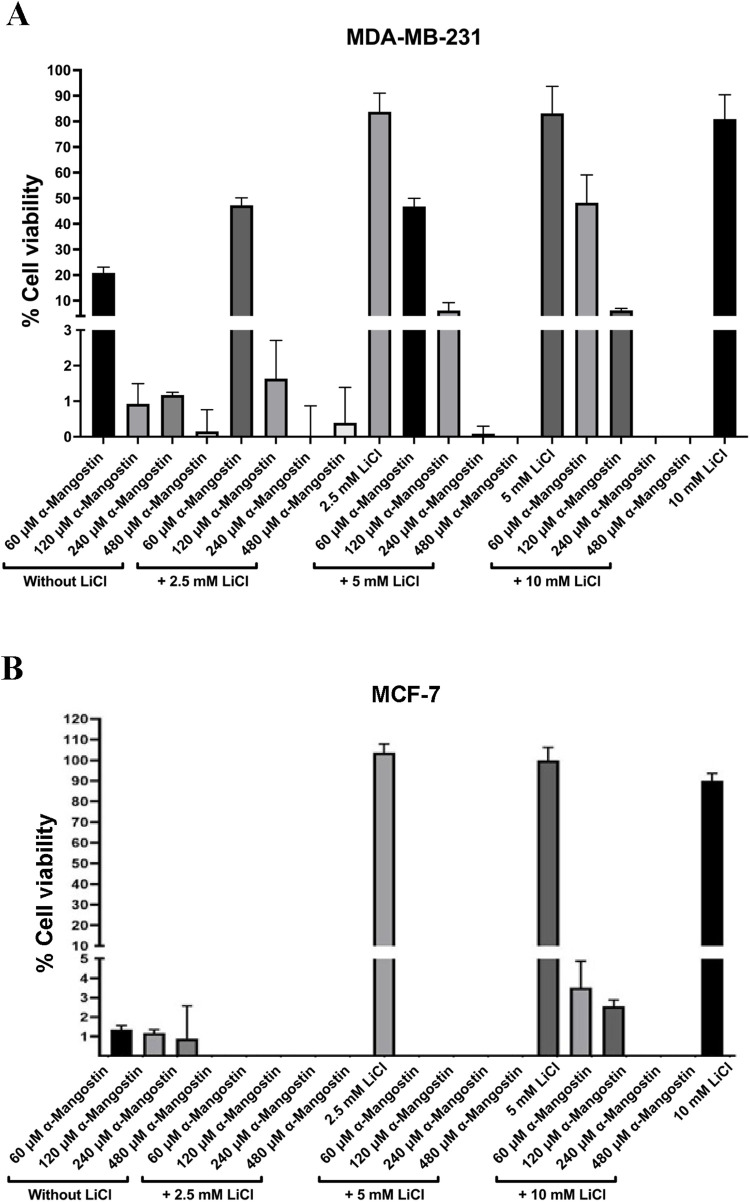
The antiproliferative effect of α-mangostin, both alone and in combination with lithium chloride (LiCl), on **(A)** MDA-MB-231 and **(B)** MCF-7 breast cancer cells. Data are presented as the mean of three independent experiments ±SD.

In the proliferation assay with MDA-MB-231 cells, α-mangostin was tested at concentrations ranging from 60 to 480 µM in combination with LiCl to activate Wnt signaling. After 24 h of treatment, α-mangostin markedly suppressed cell viability, reducing survival to 20% even at the lowest concentration (60 µM), thereby demonstrating a strong growth-inhibitory effect. In contrast, treatment with LiCl alone at varying concentrations did not significantly affect cell viability, which remained above 80%. Notably, co-treatment with α-mangostin and LiCl further reduced cell viability (Figure 6A), indicating that α-mangostin effectively suppressed the growth of MDA-MB-231 cells irrespective of Wnt activation status. A similar effect was observed in MCF-7 cells, where α-mangostin displayed even greater potency, markedly reducing viability at the lowest tested concentration (60 µM). The cytotoxic effect was further enhanced when cells were co-treated with LiCl (Figure 6B). However, because MCF-7 cells exhibited extremely low viability under these treatment conditions, further molecular analyses could not be performed on this cell line. Instead, subsequent investigations focused on the TNBC MDA-MB-231 cell line, in which the impact of α-mangostin on Wnt target gene transcription could be more reliably evaluated. Furthermore, since aberrant Wnt signaling is more prominently associated with TNBC (Pohl et al., 2017), this focus on MDA-MB-231 cells was considered well justified.

### 3.3 Antiproliferative effect of α-mangostin correlates with *CCND1* and *MYC* transcription levels in breast cancer cells

**FIGURE 7:**
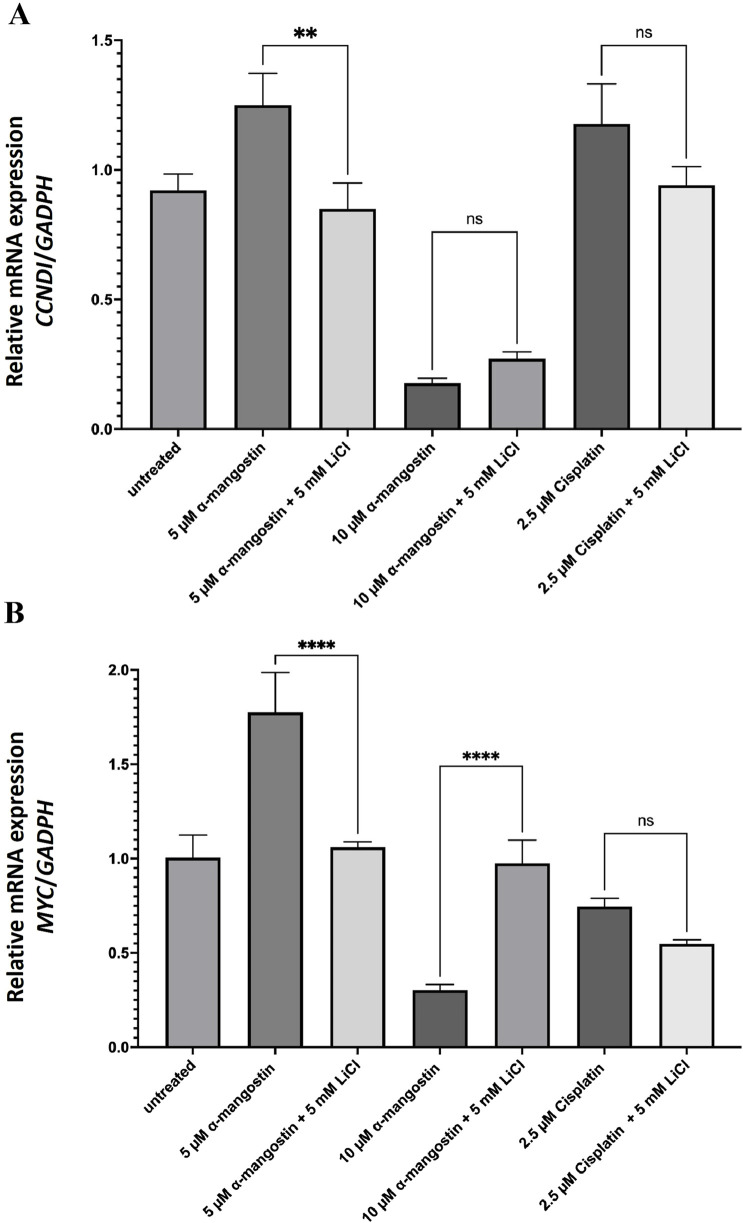
The mRNA expression levels of **(A)** *CCND1* and **(B)** *MYC* in LiCl-induced MDA-MB-231 cells following treatment with α-mangostin. Data represent the mean ± standard deviation (SD) of three independent experiments. Statistical significance: **p < 0.01; ***p < 0.001; ns, not significant.

Based on *in silico* affinity test results, α-mangostin exhibited a strong binding affinity for β-catenin, which may contribute to its degradation. Consistently, cell proliferation inhibition assays revealed that α-mangostin significantly reduced the viability of MDA-MB-231 cells. To further clarify the underlying mechanism, we performed subsequent analyses on MDA-MB-231 cells and measured the expression of proliferation-related genes regulated by Wnt signaling, specifically *CCND1* and *MYC* (Sanjari et al., 2020). Wnt signaling was activated using LiCl, which inhibits GSK-3β-mediated phosphorylation of β-catenin (Park et al., 2020). This inhibition prevents β-catenin degradation, allowing it to accumulate in the nucleus and activate transcription factors that promote cell proliferation. Our results revealed that 10 µM α-mangostin significantly suppressed *CCND1* transcription in both the absence and presence of LiCl, with a 5.2-fold reduction compared to untreated cells (Figure 7A). For comparison, we also evaluated *CCND1* expression following cisplatin treatment, as alterations in this gene are linked to chemoresistance; however, cisplatin did not produce a significant effect on *CCND1* transcription. Similarly, α-mangostin (10 µM) reduced *MYC* mRNA levels by 3.3-fold relative to the untreated group, independent of LiCl treatment (Figure 7B). Collectively, these findings suggest that α-mangostin exerts antiproliferative effects in MDA-MB-231 cells by modulating the transcription of key Wnt-regulated genes involved in proliferation.

## 4 Discussions

The Wnt/β-catenin signaling pathway plays a central role in the pathogenesis of multiple cancers, including TNBC, where its aberrant activation promotes tumor progression, metastasis, and resistance to chemotherapy (Pohl et al., 2017; Yang et al., 2022; Yang Z. et al., 2021). Under normal conditions, β-catenin is regulated by glycogen synthase kinase-3β (GSK-3β), which phosphorylates β-catenin and targets it for ubiquitination and subsequent proteasomal degradation (Shang et al., 2017). In TNBC, however, dysregulation of this regulatory mechanism results in β-catenin stabilization and nuclear translocation, where it activates oncogenic targets such as *CCND1* (*Cyclin D1*) and *MYC* (*c-Myc*), thereby driving uncontrolled cell proliferation (Dey et al., 2013; Kafri et al., 2016; Xu et al., 2016). Although β-catenin represents an attractive therapeutic target, no clinically approved drugs are currently available that directly and selectively inhibit this pathway (Morris et al., 2022). This limitation highlights the need for alternative therapeutic strategies, including the exploration of natural compounds that have the potential to modulate Wnt/β-catenin signaling.

Among natural compounds, α-mangostin has been reported to exert broad-spectrum anticancer effects; however, its direct impact on the Wnt/β-catenin pathway in TNBC remains insufficiently characterized. The present study provides new evidence showing that α-mangostin significantly suppresses the proliferation of MDA-MB-231 cells, even in the presence of Wnt pathway activation by LiCl. This suggests that α-mangostin exerts cytotoxic effects independently of β-catenin stabilization, distinguishing it from conventional Wnt-targeting agents. Our results are consistent with those of Yoo et al. (2011), in colon cancer cells, α-mangostin disrupted Wnt/β-catenin signaling by reducing β-catenin expression at both the mRNA and protein levels. Notably, this reduction occurred without initiating the canonical degradation process of β-catenin and was independent of β-catenin mutation status. Furthermore, LiCl treatment, which activates Wnt signaling, did not diminish the inhibitory effects of α-mangostin, suggesting that its mechanism of action bypasses the conventional β-catenin degradation pathway. Similarly, in our study, α-mangostin modulated the transcription of downstream Wnt target genes without directly affecting β-catenin degradation, further supporting the hypothesis of an alternative mechanism of pathway suppression.

Additionally, α-mangostin demonstrated greater cytotoxic sensitivity in MCF-7 cells, reinforcing its strong antiproliferative effects in luminal breast cancer. Previous studies have attributed its activity in MCF-7 cells to the induction of mitochondrial-mediated apoptosis through Bax oligomerization, cytochrome c release, and caspase activation (Simon et al., 2022). Consistently, Nalla et al. (2023) reported that α-mangostin suppressed Ki-67 expression, inhibited cell migration by modulating EMT markers such as MMP-2 and PKM-2, and downregulated STAT3 activation (Nalla et al., 2023). Furthermore, both α-mangostin and γ-mangostin were shown to inhibit the migration of MDA-MB-231 cells via transcriptional suppression of CXCR4, underscoring their multi-targeted capacity in attenuating breast cancer progression (Sarmoko et al., 2023).

**FIGURE 8:**
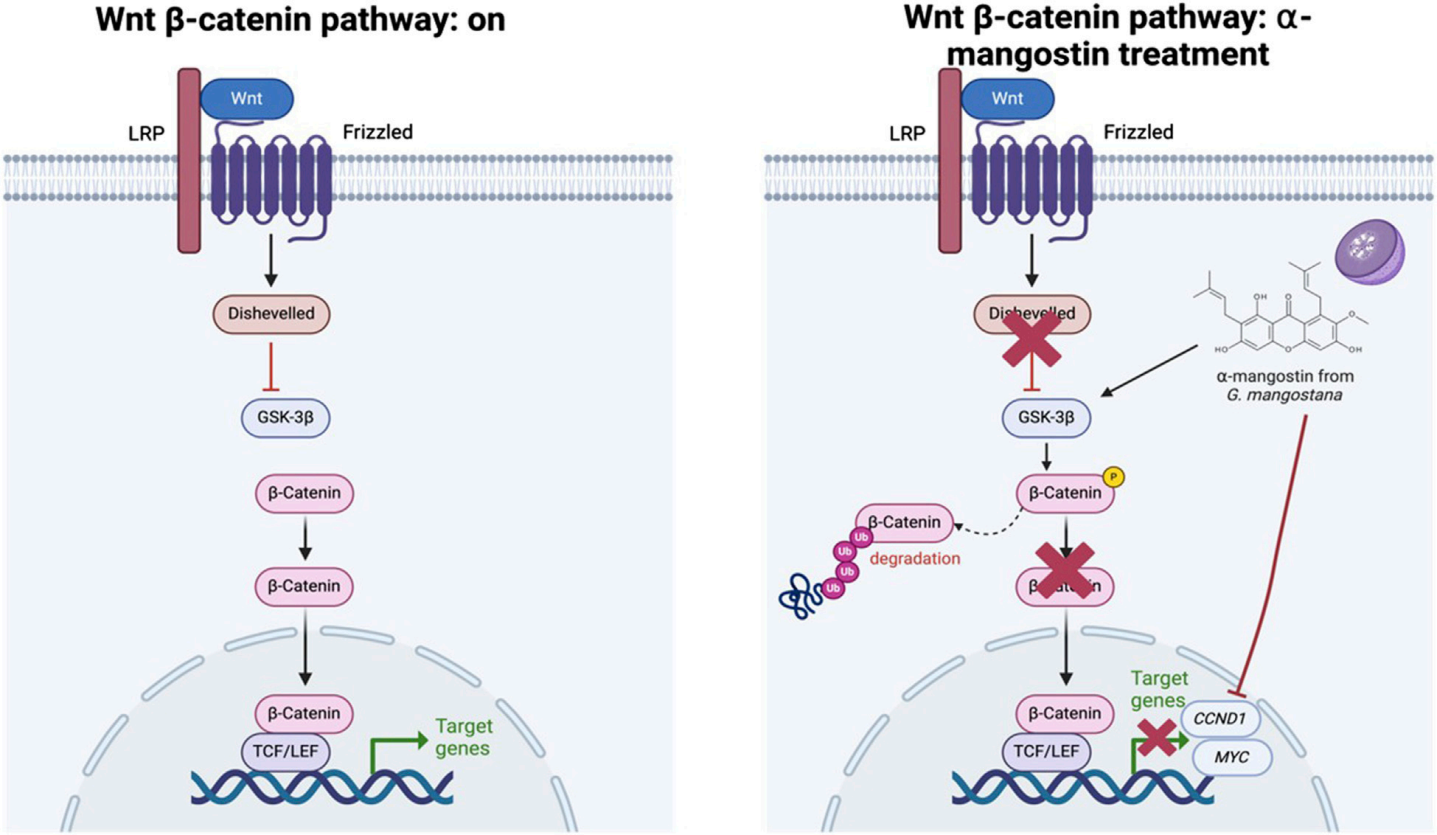
Proposed mechanism of action of α-mangostin in modulating the Wnt/β-catenin signaling pathway.

Molecularly, our results confirm that α-mangostin significantly downregulated *CCND1* and *MYC* expression in MDA-MB-231 cells, irrespective of LiCl-mediated Wnt activation. This observation is consistent with previous reports indicating that α-mangostin inhibits TCF/LEF transcriptional activity by promoting β-catenin degradation, thereby reducing the expression of Wnt target genes (Zhu et al., 2021), as illustrated in Figure 8. Notably, LiCl treatment did not reverse the β-catenin degradation induced by α-mangostin, suggesting that its mechanism may bypass the classical Wnt/β-catenin signaling cascade (Yoo et al., 2011). Furthermore, cisplatin, a widely used chemotherapeutic agent, had no significant effect on *CCND1* expression in our study, supporting the notion that prolonged cisplatin exposure contributes to Wnt-mediated chemoresistance in TNBC (Yin et al., 2013).

Beyond its role in cell cycle regulation, α-mangostin has also been shown to modulate intracellular reactive oxygen species (ROS) levels, triggering endoplasmic reticulum (ER) stress in a Wnt-dependent manner. In osteosarcoma cells, α-mangostin inhibited GSK-3β activity, leading to ROS-induced ER stress and blocking β-catenin nuclear translocation (Yang S. et al., 2021). Similarly, an increase in ROS levels was observed in MDA-MB-231 cells treated with α-mangostin (Sarmoko et al., 2023), despite the well-documented antioxidant properties of this compound. This apparent dual role, acting as both pro-oxidant and antioxidant, is reminiscent of polyphenols such as curcumin, which exhibit context-dependent oxidative modulation in cancer cells (Wolnicka-Glubisz and Wisniewska-Becker, 2023). Considering these dual effects, it is important to recognize their implications when developing rational strategies for natural compound-based cancer therapeutics. The ability of α-mangostin to selectively induce oxidative stress in malignant cells while sparing normal cells underscores its therapeutic potential. Harnessing this biphasic behavior could provide a foundation for more effective and targeted anticancer strategies, potentially minimizing drug resistance and improving treatment outcomes.

Our molecular docking and simulation analyses indicate that α-mangostin modulates Wnt/β-catenin signaling by targeting both β-catenin and LRP6. Since β-catenin is central to oncogenic transcription, particularly when exacerbated by APC mutations (Hankey et al., 2018), and LRP6 serves as an upstream Wnt co-receptor, the stronger interaction of α-mangostin with LRP6 suggests its potential to block Wnt signaling at early stages of pathway activation. This dual-inhibition strategy, suppressing signaling at multiple levels (Raisch et al., 2019), could mitigate drug resistance often associated with single-target therapies, thereby improving overall treatment efficacy (Raisch et al., 2019; Ariyanto et al., 2023).

The absence of clinically approved selective Wnt/β-catenin inhibitors, despite the pathway’s involvement through components such as β-catenin, GSK-3β, Axin, APC, and LRP6, underscores the therapeutic relevance of natural compounds like α-mangostin. Our earlier *in silico* findings suggested that α-mangostin may inhibit GSK-3β by modulating the Wnt/β-catenin pathway, although this had not been validated *in vitro*. To address this, the present study performed comparative *in silico* analyses, docking α-mangostin and the reference Wnt inhibitor XAV939 to LRP6, followed by molecular dynamics simulations and MM/PBSA binding energy calculations. Consistently, α-mangostin demonstrated a more favorable interaction profile with LRP6, as reflected by more negative binding free energy and enhanced structural stability. These results strengthen the hypothesis that α-mangostin targets LRP6 to modulate Wnt signaling. While experimental validation using LRP6-specific inhibitors or knockdown approaches was beyond the scope of this study, it remains a critical direction for future work, which could be pursued using techniques such as Western blotting or β-catenin luciferase reporter assays (Lai et al., 2009). Taken together, our findings support α-mangostin as a promising therapeutic candidate for aggressive and therapy-resistant breast cancer subtypes. Unlike conventional Wnt-targeting therapies, α-mangostin exerts its effects independently of β-catenin stabilization, making it a potentially more versatile treatment option.

A similar phenomenon was reported with EGCG in breast cancer cells, where β-catenin expression remained unchanged while *MYC* transcription was suppressed (Kim et al., 2006). Likewise, the polyphenol curcumin inhibited Wnt signaling by preventing β-catenin nuclear translocation, thereby downregulating downstream targets such as *CCND1* and *MYC* (Prasad et al., 2009). Given its multi-target effects, including regulation of the cell cycle, induction of apoptosis, and inhibition of metastasis, further *in vitro* and *in vivo* investigations are warranted to establish α-mangostin as a potential therapeutic agent.

Although drug development targeting the Wnt signaling pathway in cancer has made promising progress, no specific Wnt-targeted therapy has yet been approved for clinical use. This remains a significant challenge in advancing therapeutic strategies targeting the canonical Wnt/β-catenin pathway. Small-molecule inhibitors, both synthetic and naturally derived, often display favorable bioavailability and cellular permeability; however, concerns about off-target effects persist (Yu et al., 2021). These concerns arise primarily from the essential role of the Wnt/β-catenin pathway in normal physiological processes, making the selective targeting of this pathway inherently difficult. In addition, the scarcity of well-defined druggable structures within Wnt/β-catenin signaling components further complicates the development of selective and effective inhibitors, thereby limiting their clinical translation (Cui et al., 2018). Addressing these obstacles will require a comprehensive evaluation of existing compounds and the design of novel strategies, such as combination approaches or synergistic therapeutic regimens, which may ultimately enable the successful development of effective Wnt-targeted cancer treatments.

Future investigations should prioritize evaluating the synergistic potential of α-mangostin in combination with established chemotherapeutic agents to improve treatment outcomes and mitigate resistance. Furthermore, a detailed exploration of the molecular interactions between α-mangostin, GSK-3β, and β-catenin will be essential to clarify its precise role in modulating Wnt signaling. Preclinical validation using TNBC animal models will also be critical to confirm its therapeutic efficacy under physiological conditions. By uncovering a novel mechanism of action, this study provides a foundation for the potential clinical application of α-mangostin as an anti-TNBC agent. With continued research, α-mangostin may emerge as a promising addition to the current repertoire of breast cancer therapeutics, offering new opportunities for patients with limited treatment options.

## 5 Conclusion

Our study demonstrated that α-mangostin suppresses the transcription of *CCND1* (cyclin D1) and *MYC* (c-Myc) in Wnt-activated MDA-MB-231 TNBC cells. Computational analyses further revealed that α-mangostin binds at sites overlapping with LRP6 inhibitors, suggesting its potential to disrupt the LRP6–β-catenin complex. Together, these findings highlight a novel mechanism by which α-mangostin may modulate the Wnt/β-catenin signaling pathway. Future studies should investigate the synergistic potential of α-mangostin in combination with established chemotherapies to enhance therapeutic outcomes and overcome resistance. In addition, a more profound exploration of its molecular interactions with GSK-3β and β-catenin will be essential to define further its role in regulating this pathway.

## Data Availability

The original contributions presented in the study are included in the article/supplementary material, further inquiries can be directed to the corresponding author.
